# Microbiome-innate immune crosstalk in acute exacerbation of idiopathic pulmonary fibrosis: an amplification framework

**DOI:** 10.3389/fimmu.2026.1847027

**Published:** 2026-05-01

**Authors:** Wanwan Zhang, Jiayang Yi, Zhi Li

**Affiliations:** 1 Department of Pulmonary and Critical Care Medicine, The First Affiliated Hospital of Wannan Medical University, Wuhu, Anhui, China; 2 Department of Rheumatology, The First Affiliated Hospital of Wannan Medical University, Wuhu, Anhui, China

**Keywords:** AE-IPF, biofilm, innate immunity, lung microbiome, macrophages, NEtosis, NLRP3 inflammasome

## Abstract

Acute exacerbation of idiopathic pulmonary fibrosis (AE-IPF) remains a major cause of short-term mortality, yet its biological drivers are incompletely understood. Increasing evidence indicates that the distal lung is not sterile and that microbial burden, ecological disruption, and host immune responses may interact within the fibrotic niche. Here, we summarize current evidence and propose a focused, hypothesis-generating framework for microbiome-immune crosstalk in AE-IPF. In this model, pulmonary dysbiosis and persistent microbial stimulation may converge with epithelial injury-derived danger signals to amplify pattern-recognition receptor signaling, reshape macrophage states, and promote injurious neutrophil responses, including excessive neutrophil extracellular trap formation. Emerging data from fibrotic lung disease also raise the possibility that SPP1-associated macrophage programs may contribute to both impaired host defense and maladaptive repair. These observations also support the view that immune-cell state transitions, rather than microbial burden alone, may shape how acute injury is translated into persistent fibroinflammatory remodeling. We also discuss how antimicrobial exposure and resistance-associated persistence traits, including biofilm formation, could contribute to impaired microbial clearance and sustained innate immune activation, while emphasizing that direct *in situ* evidence in AE-IPF remains limited. Rather than proposing a universal mechanism, this mini review highlights a testable amplification framework that may help refine pathogen-aware stratification, antimicrobial stewardship, and biomarker-guided host-directed strategies in future studies.

## 1 Introduction

Idiopathic pulmonary fibrosis (IPF) is a progressive fibrotic interstitial lung disease characterized by irreversible remodeling of the distal lung and progressive respiratory failure (1–4). Acute exacerbation of IPF (AE-IPF) marks a critical inflection point in the disease course and remains a major determinant of short-term mortality (5). Although antifibrotic agents such as nintedanib and pirfenidone slow the decline in stable IPF, their benefit during an established AE-IPF event is limited (6, 7). As a result, corticosteroids and empiric immunomodulatory strategies are still widely used despite uncertain efficacy and concern for infectious complications (8, 9).

Culture-independent sequencing has challenged the historical assumption that the lower airways are sterile. Instead, the distal lung appears to contain a low-biomass microbial community shaped by microaspiration, mucociliary clearance, local immune surveillance, and structural lung integrity (10, 11). In IPF, this ecological balance appears to be disturbed, and higher microbial burden has been associated with worse clinical outcomes (12, 13). Although such observations do not establish causality, they justify closer examination of microbiome-host interactions in fibrotic lung disease.

This review argues that AE-IPF should not be framed solely as “sterile inflammation” or “superimposed infection.” Rather, in a subset of susceptible patients, pulmonary ecological disruption, persistent microbial stimulation, antimicrobial selection pressure, and maladaptive immune signaling may together form a self-reinforcing amplification loop (6, 14). The goal of this framework is not to propose a universal mechanism, but to distinguish clinical associations, biologically plausible inferences, and cross-disease extrapolations that may guide future mechanistic and translational studies.

## 2 Dynamic shifts of the lung microbiome in AE-IPF

### 2.1 From low-biomass homeostasis to dysbiosis

Interpreting pulmonary microbiome data in IPF requires stringent methodological control, because upper-airway contamination, low-biomass artifacts, sampling differences, and bioinformatic thresholds can all influence signal detection (15–18). With these caveats in mind, available 16S rRNA and metagenomic studies suggest disruption of lower-airway ecological homeostasis in IPF rather than a single disease-defining microbial signature (19, 20).

Healthy distal airways appear enriched in relatively tolerogenic commensals, including *Prevotella* (21), whereas IPF cohorts often show increased bacterial burden, relative enrichment of *Firmicutes* and *Proteobacteria* (22–24), and depletion of taxa associated with immune homeostasis. Immunologically, the expansion of Gram-negative *Proteobacteria* introduces abundant lipopolysaccharide (LPS), providing persistent Toll-like receptor 4 (TLR4)-mediated signals that can chronically prime the alveolar immune niche (12, 13). These observations are consistent with a microenvironment that may favor inflammatory signaling, although functional and metabolic features appear more reproducible than any individual taxon.

### 2.2 Microbial burden, dominant taxa, and outcome associations

In stable IPF, higher bacterial burden in bronchoalveolar lavage fluid (BALF) has been associated with worse lung function, disease progression, and mortality (25, 26). Taxa such as *Streptococcus*, *Staphylococcus*, and *Haemophilus* are recurrent associative signals (27), although none can currently be considered a universal disease driver. Dedicated AE-IPF datasets remain limited and are frequently confounded by hospitalization, prior antimicrobial exposure, aspiration, and disease severity (27).

Nevertheless, available observations are compatible with a further increase in microbial burden during acute events in at least a subset of patients. In this context, *Pseudomonas aeruginosa* is best viewed as a model pathogen for multidrug resistance and biofilm-associated persistence rather than as a proven ubiquitous trigger of AE-IPF.

## 3 A working model of microbiome-immune amplification in AE-IPF

**Figure 1:**
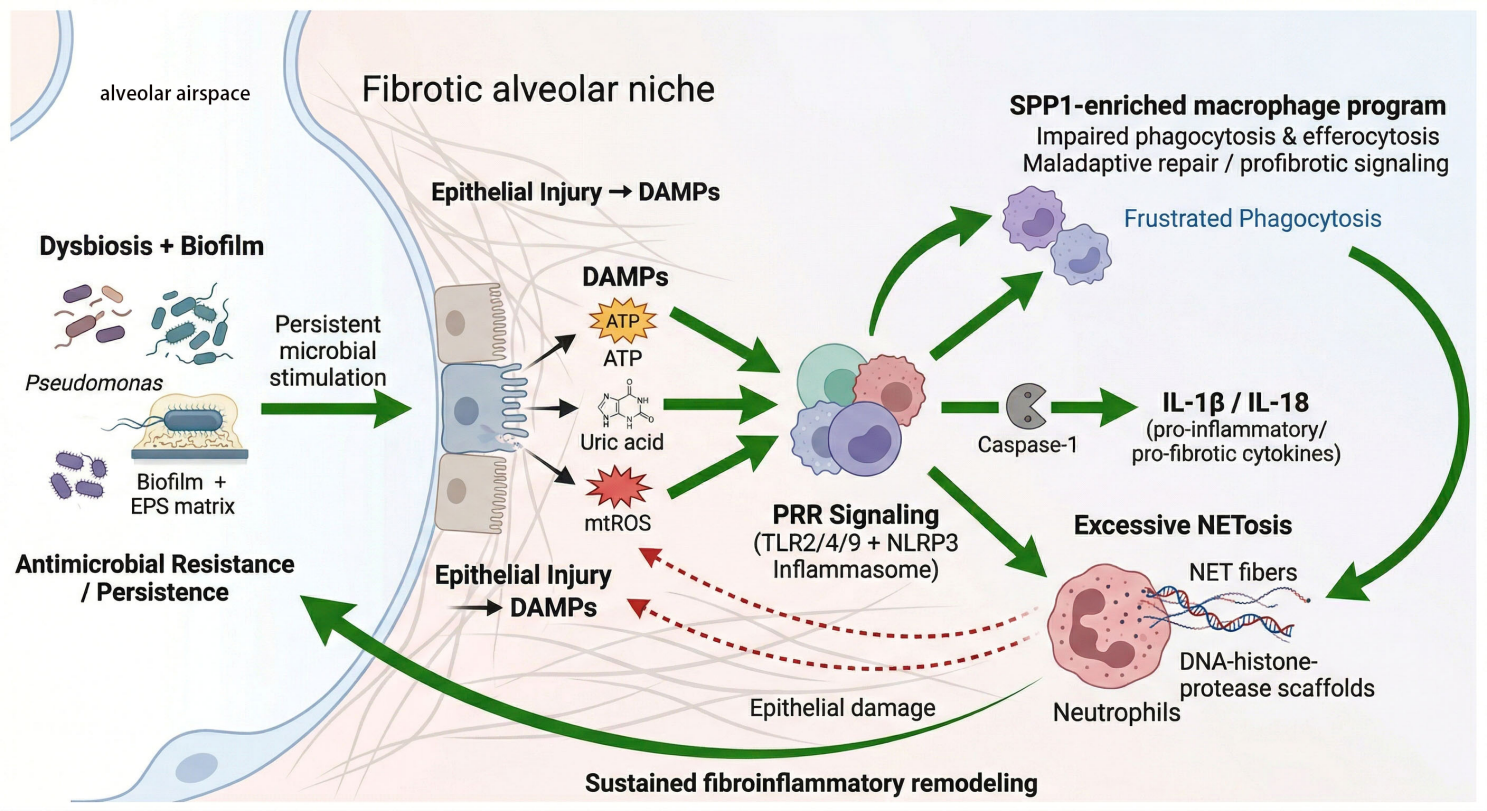
A testable amplification framework of microbiome-immune crosstalk and antimicrobial persistence in acute exacerbation of idiopathic pulmonary fibrosis (AE-IPF). Within the structurally vulnerable fibrotic alveolar niche, persistent microbial stimulation within the alveolar airspace—driven by dysbiosis and biofilm formation (e.g., *Pseudomonas* enclosed in an extracellular polymeric substance [EPS] matrix)—converges with endogenous damage-associated molecular patterns (DAMPs; e.g., extracellular ATP, uric acid, and mitochondrial ROS [mtROS]) released from the pre-injured epithelium. This dual stimulation massively amplifies pattern-recognition receptor (PRR) signaling, predominantly via Toll-like receptors (TLR2/4/9) and the NLRP3 inflammasome, culminating in caspase-1-dependent secretion of pro-inflammatory and pro-fibrotic cytokines (IL-1β and IL-18). Downstream, this robust innate activation drives two maladaptive effector pathways (1): the emergence of SPP1-enriched macrophage programs characterized by impaired phagocytosis/efferocytosis and pro-fibrotic signaling, and (2) excessive neutrophil extracellular trap (NET) formation. Furthermore, structurally resilient biofilms physically shield pathogens, inducing “frustrated phagocytosis” and contributing to antimicrobial resistance. Crucially, the toxic DNA-histone-protease scaffolds from NETosis provoke secondary epithelial damage (red dashed arrows), releasing additional DAMPs. Together, these elements form a self-reinforcing vicious cycle linking impaired microbial clearance to sustained fibroinflammatory remodeling. (AE-IPF, acute exacerbation of idiopathic pulmonary fibrosis; ATP, adenosine triphosphate; CitH3, citrullinated histone H3; DAMP, damage-associated molecular pattern; EPS, extracellular polymeric substance; HMGB1, high-mobility group box 1; IL, interleukin; mtROS, mitochondrial reactive oxygen species; NET, neutrophil extracellular trap; NF-κB, nuclear factor kappa B; NLRP3, NLR family pyrin domain containing 3; PAD4, peptidylarginine deiminase 4; PRR, pattern-recognition receptor; SPP1, secreted phosphoprotein 1; TLR, Toll-like receptor).

Based on the synthesis of current evidence, we propose a multi-cellular amplification framework wherein persistent microbial stimulation, epithelial injury, and maladaptive innate immune responses interact within the fibrotic alveolar niche (**Figure 1**). In this self-reinforcing loop, antimicrobial persistence mechanisms and injury signals converge to reshape local macrophage and neutrophil states, ultimately driving persistent fibroinflammatory remodeling.

### 3.1 A pre-injured alveolar epithelium as the permissive context

The IPF alveolar unit is structurally and immunologically vulnerable (28, 29). Epithelial fragility, altered barrier integrity, distorted architecture, impaired clearance, and aberrant regenerative responses create a niche in which relatively modest microbial or injury-derived signals may provoke disproportionate inflammation (20, 30, 31). In AE-IPF, sustained microbial exposure may lower the threshold for innate immune activation, while epithelial apoptosis and necrosis release endogenous alarmins that can further amplify local injury responses (6).

### 3.2 PAMP-DAMP convergence and PRR signaling

Within the dysbiotic fibrotic lung, pathogen-associated molecular patterns (PAMPs) and damage-associated molecular patterns (DAMPs) may converge on overlapping pattern-recognition receptor (PRR) networks, including Toll-like receptor (e.g., TLR2, TLR4, TLR9) networks and specific inflammasome complexes, predominantly the NLRP3 inflammasome (**Figure 1**). In the fibrotic niche, microbial PAMPs can provide the essential ‘priming’ signal (Signal 1) via TLRs to drive NF-κB-dependent transcription of pro-IL-1β and inflammasome components, while tissue injury-derived DAMPs—such as extracellular ATP, uric acid, high-mobility group box 1 (HMGB1), or mitochondrial ROS from necrotic epithelium—serve as secondary ‘activation’ signals (Signal 2) for NLRP3 oligomerization. This robust innate convergence culminates in caspase-1 activation and the release of potent pro-inflammatory and pro-fibrotic cytokines, including IL-1β and IL-18, thereby amplifying both sterile and microbe-associated inflammation (32–34). Importantly, activation of these pathways should not be interpreted as proof of overt infection, because similar signaling can also arise from injury-derived alarmins alone (32).

Potential host susceptibility factors proposed in IPF, including altered TLR3 signaling and dysregulated cytosolic DNA sensing, may further shape the magnitude and quality of these responses (35, 36). In addition, excessive PRR activation may intersect with inflammatory cell-death programs and sustained epithelial stress, thereby reinforcing DAMP release within an already fragile alveolar niche (37). However, the relative contribution of these pathways during AE-IPF remains incompletely defined (36, 38). Taken together, these observations are consistent with the possibility that, in at least a subset of patients, AE-IPF involves both impaired microbial control and excessive injury signaling rather than a simple state of generalized immune activation (39). From an immunologic perspective, this framework suggests that AE-IPF may reflect a failure to appropriately compartmentalize microbial sensing, tissue-damage signaling, and resolution programs within the injured alveolar niche (39).

### 3.3 Macrophage remodeling and emergence of SPP1-associated phenotypes

Alveolar macrophages are central regulators of microbial sensing, efferocytosis, and tissue repair (40). Single-cell studies in IPF indicate depletion of homeostatic tissue-resident alveolar macrophages and their replacement, at least in part, by recruited monocyte-derived macrophages (41, 42). Within the fibrotic niche, these cells may acquire SPP1-enriched programs associated with altered tissue-remodeling functions and reduced homeostatic features (43).

Although direct evidence from AE-IPF remains limited, macrophage remodeling may represent one plausible link between defective host defense and maladaptive repair. In this context, SPP1-associated macrophage programs may be viewed not only as profibrotic cell states, but also as potential immune-metabolic intermediates linking impaired clearance to persistent stromal activation (44, 45). On the one hand, SPP1-associated macrophage states may drive fibroblast activation, matrix deposition, and maintenance of a profibrotic microenvironment (46, 47). On the other hand, impaired phagocytosis or efferocytosis could favor retention of microbial ligands, dead-cell material, and unresolved inflammatory cues (48). Emerging data from fibrotic lung disease also suggest that these macrophage states may be shaped by local metabolic stress and niche-specific signals (44, 45), but the relevance of such programs to acute exacerbation requires further study. Thus, macrophage dysfunction in AE-IPF is best viewed as a plausible amplifier of both ecological disruption and tissue injury rather than as a fully established disease-defining mechanism (46).

### 3.4 NETosis as a double-edged effector mechanism

Neutrophil extracellular trap (NET) formation may initially contribute to microbial containment (49), but excessive or persistent NETosis can become tissue damaging (50). Driven by peptidylarginine deiminase 4 (PAD4)-dependent chromatin decondensation, NET-associated citrullinated histones (e.g., CitH3), proteases, and chromatin scaffolds may worsen epithelial injury, amplify local cytokine release, and expose matrix and self-antigens within already fragile distal airways (51) (**Figure 1**, red dashed arrows).

In fibrotic microenvironments, dense extracellular matrix, altered clearance, and ongoing myeloid activation may be associated with prolonged NET persistence even when the initiating insult is no longer dominant (52, 53). This raises the possibility that NETosis is not only a consequence of acute innate activation, but may also contribute to the persistence of tissue injury after transient triggers. Some experimental and cross-disease observations further suggest that matrix-rich environments may shape the mode and persistence of NET release (54), although the relative contribution of distinct NETotic programs in AE-IPF remains uncertain. Accordingly, NETosis may be one biologically plausible contributor linking microbial stimulation, epithelial injury, and subsequent profibrotic remodeling, while its downstream effects on adaptive immunity in AE-IPF remain incompletely defined (54, 55). Consequently, NET persistence may be particularly relevant in AE-IPF not simply as a marker of neutrophil activation, but as a candidate interface through which innate immune injury is prolonged and potentially propagated across other immune compartments.

### 3.5 Innate-to-adaptive immune crosstalk and tolerance breakdown

Compared with innate immune pathways, adaptive immune alterations in AE-IPF are much less well characterized. Data from stable IPF and related fibrotic settings suggest that T-cell dysfunction, altered regulatory-effector balance, and B-cell remodeling may contribute to disease progression in a subset of patients (39, 56), but their causal role during acute exacerbation remains uncertain.

Driven by microbiota-induced PRR activation, the local IL-6- and TGF-β-rich milieu may strongly favor the expansion of Th17 cells, which in turn secrete IL-17 to sustain local neutrophil recruitment (57). Concurrently, the excessive NETosis discussed above can expose chromatin scaffolds and modified self-antigens—particularly proteins citrullinated by PAD4 during NET formation—to the adaptive immune system, acting as a crucial bridge between innate tissue injury and adaptive auto-reactivity. This NET-driven autoantigen presentation can break peripheral immune tolerance, leading to aberrant B-cell activation and autoantibody production, as described in fibrotic lung disease more broadly (55). These observations raise the possibility that adaptive immune circuits may reinforce epithelial injury and innate immune activation once an exacerbation has been initiated (58). However, mechanistic models linking microbial nucleic acid sensing, loss of immune tolerance, and pathogenic humoral activation in AE-IPF remain preliminary and should currently be regarded as hypothesis-supporting rather than disease-defining evidence. Even if these adaptive pathways are not primary initiators of AE-IPF, they may still influence whether an acute inflammatory episode resolves, propagates, or transitions toward sustained fibroinflammatory remodeling.

## 4 Antimicrobial resistance, microbial persistence, and the fibrotic niche

Empirical broad-spectrum antimicrobial exposure during AE-IPF may further perturb an already unstable microbial ecosystem and increase selection pressure for resistant organisms (15, 18, 59). In this context, antimicrobial resistance is not only a pharmacologic problem but also a potential ecological driver of persistence within the fibrotic niche.

One plausible persistence mechanism is biofilm formation. Organisms such as *Pseudomonas aeruginosa* or *Staphylococcus* aureus are useful model pathogens because biofilm-associated growth may impair immune clearance (60), prolong myeloid cell activation (61, 62), and sustain local inflammatory signaling. A related hypothesis is that biofilm-associated “frustrated phagocytosis” occurs when the bacterial extracellular polymeric substance (EPS) matrix physically shields pathogens from complement deposition, antimicrobial peptides, and efficient opsonic engulfment. This structural resistance forces recruited myeloid cells into a state of continuous, fruitless degranulation and reactive oxygen species (ROS) overproduction. Crucially, this localized oxidative stress and subsequent lysosomal destabilization (e.g., cathepsin release) serve as potent, direct secondary triggers for robust NLRP3 inflammasome hyperactivation (63). This sustains macrophage activation in the absence of effective microbial clearance (**Figure 1**, frustrated phagocytosis), thereby locking the fibrotic niche in a state of persistent PRR engagement and profibrotic cytokine release (64). In this framework, resistance and persistence should be viewed as overlapping rather than entirely separate biological problems. Under this view, resistance-associated persistence traits may be immunologically important less because they increase pathogen abundance *per se* than because they prolong antigenic and innate stimulatory pressure within a structurally vulnerable lung.

At the same time, direct evidence for mature polymicrobial biofilms within distal fibrotic regions in AE-IPF remains limited (18), and this link should currently be regarded as testable rather than established. Broad antimicrobial exposure and immune disequilibrium may also permit fungal overgrowth or latent viral reactivation in selected patients (65). Yet co-detection should not be equated with pathogenic contribution (6, 66), and the frequency and mechanistic relevance of such inter-kingdom signals in AE-IPF remain unresolved (39, 67).

## 5 Discussion: controversies, evidence gaps, and translational priorities

In the near term, the most actionable implication of this framework may be better pathogen-aware stratification combined with antimicrobial stewardship (68). Within the context of microbiome-innate immune crosstalk, while empiric broad-spectrum antibiotics are widely used, they may inadvertently perpetuate the inflammatory amplification loop by further disrupting the fragile lung ecology and selecting for resistance-associated persistence phenotypes. To optimize therapeutic strategies, BALF molecular microbiology, targeted polymerase chain reaction panels, or metagenomic profiling may help distinguish likely pathogens from ecological noise and reduce unnecessary broad-spectrum exposure when interpreted within the clinical context. Consequently, this approach would facilitate a shift toward targeted, narrow-spectrum antimicrobial therapies. Such precision strategies could effectively clear specific microbial triggers while minimizing collateral ecological damage, ultimately helping to break the cycle of sustained innate immune activation and fibrotic remodeling.

A second translational direction is host endotyping (69, 70). If reproducible immune-microbial endotypes can be identified, candidate host-directed approaches could include modulation of profibrotic innate signaling, attenuation of NET-associated injury, and selective targeting of maladaptive inflammatory pathways (**Table 1**). Emerging antifibrotic and immunomodulatory strategies in fibrotic lung disease further underscore the importance of identifying biologically distinct patient subsets that may inform microbiome-aware and biomarker-guided approaches, rather than treating AE-IPF as a biologically uniform event.

**Table 1:** Key biological nodes of the microbiome-innate immune amplification loop in AE-IPF and their translational implications.

Biological compartment/Process	Key molecular mediators & signatures	Pathogenic contribution to the amplification loop	Potential host-directed or translational strategies
Microbial Dysbiosis & Biofilms	Expansion of *Proteobacteria* (LPS), Pseudomonas, EPS matrix	Provide persistent TLR4 priming (Signal 1); biofilms shield pathogens and induce “frustrated phagocytosis.”	Pathogen-aware stratification (e.g., BALF mNGS); precision antimicrobial stewardship; biofilm-disrupting agents.
Pre-injured Alveolar Epithelium	Endogenous DAMPs (extracellular ATP, HMGB1, mtROS, uric acid)	Act as secondary ‘activation’ signals (Signal 2), lowering the threshold for robust inflammasome assembly.	Alarmin-neutralizing therapies (e.g., anti-HMGB1 antibodies); DAMP scavengers; cytoprotective targeted agents.
PRR & Inflammasome Convergence	TLR2/4/9, NLRP3 inflammasome, Caspase-1, IL-1β, IL-18	Synergistically amplify sterile and microbe-associated inflammation; drive massive pro-fibrotic cytokine cascades.	Specific small-molecule NLRP3 inhibitors; selective TLR signaling blockade; IL-1 or IL-18 receptor antagonists.
Macrophage Remodeling	Emergence of SPP1-enriched macrophage programs	Impairs homeostatic efferocytosis/phagocytosis; links defective clearance to persistent extracellular matrix deposition.	Metabolic reprogramming of maladaptive macrophages; targeted blockade of the SPP1 profibrotic axis.
Excessive Neutrophil NETosis	PAD4-dependent chromatin decondensation, citrullinated histones (e.g., CitH3), toxic scaffolds	Provokes secondary epithelial damage; exposes modified autoantigens, linking innate injury to tolerance breakdown.	Pharmacological PAD4 inhibitors; inhalable polymeric nucleases (e.g., DNase) to dismantle NET scaffolds.

(AE-IPF, acute exacerbation of idiopathic pulmonary fibrosis; ATP, adenosine triphosphate; BALF, bronchoalveolar lavage fluid; CitH3, citrullinated histone H3; DAMP, damage-associated molecular pattern; EPS, extracellular polymeric substance; HMGB1, high-mobility group box 1; IL, interleukin; LPS, lipopolysaccharide; mNGS, metagenomic next-generation sequencing; mtROS, mitochondrial reactive oxygen species; NET, neutrophil extracellular trap; NLRP3, NLR family pyrin domain containing 3; PAD4, peptidylarginine deiminase 4; PRR, pattern-recognition receptor; SPP1, secreted phosphoprotein 1; TLR, Toll-like receptor).

Finally, future studies should move beyond descriptive taxonomic associations toward integrated longitudinal profiling (43, 71). Spatially resolved transcriptomics, immune phenotyping, and microbiome or resistome analysis may help determine whether microbial persistence mechanisms, macrophage remodeling, and injurious neutrophil programs truly co-localize *in situ* within the AE-IPF lung. Such approaches will be essential if the field is to distinguish driver mechanisms from epiphenomena.

## 6 Conclusion

A persistent challenge in pulmonary microbiome research is determining whether microbial enrichment acts as a primary driver, a permissive amplifier, or a secondary reflection of distorted lung architecture, aspiration, hospitalization, and prior treatment exposure. This uncertainty is especially relevant in AE-IPF, where dedicated cohort studies remain limited and mechanistic inference often relies on extrapolation from stable IPF or other chronic lung diseases.

Importantly, the framework outlined here should not be interpreted as an infection-only model of AE-IPF. Rather, it emphasizes that microbial signals may interact with epithelial injury, distorted lung architecture, and maladaptive host immunity to amplify disease trajectories in a subset of susceptible patients. Thus, the proposed microbiome-immune-antimicrobial resistance axis is best viewed as an organizing framework for generating falsifiable hypotheses rather than as a universal pathway.

Future longitudinal studies should define immune-microbial endotypes, clarify the clinical impact of resistome expansion, and determine whether microbial persistence mechanisms such as biofilm-associated host-defense failure can be validated *in situ*. Ultimately, integrating pathogen-aware stratification with biomarker-guided host immunophenotyping will likely be required if this framework is to inform future precision approaches in AE-IPF.

## Glossary

AE-IPF: acute exacerbation of idiopathic pulmonary fibrosis
ATP: adenosine triphosphate
BALF: bronchoalveolar lavage fluid
CitH3: citrullinated histone H3
DAMP: damage-associated molecular pattern
EPS: extracellular polymeric substance
HMGB1: high-mobility group box 1
IL: interleukin
IPF: idiopathic pulmonary fibrosis
LPS: lipopolysaccharide
mtROS: mitochondrial reactive oxygen species
NET: neutrophil extracellular trap
NF-κB: nuclear factor kappa B
NLRP3: NLR family pyrin domain containing 3
PAD4: peptidylarginine deiminase 4
PAMP: pathogen-associated molecular pattern
PRR: pattern-recognition receptor
ROS: reactive oxygen species
SPP1: secreted phosphoprotein 1
TGF-β: transforming growth factor-beta
Th17: T helper 17
TLR: Toll-like receptor.
