# Correction: Microbiome-innate immune crosstalk in acute exacerbation of idiopathic pulmonary fibrosis: an amplification framework

**DOI:** 10.3389/fimmu.2026.1874174

**Published:** 2026-05-12

**Authors:** Wanwan Zhang, Jiayang Yi, Zhi Li

**Affiliations:** 1 Department of Pulmonary and Critical Care Medicine, The First Affiliated Hospital of Wannan Medical University, Wuhu, Anhui, China; 2 Department of Rheumatology, The First Affiliated Hospital of Wannan Medical University, Wuhu, Anhui, China

**Keywords:** AE-IPF, biofilm, innate immunity, ung microbiome, macrophages, NEtosis, NLRP3 inflammasome

In the published article, there was an error in the **Funding** statement. The original **Funding** statement was: “The author(s) declared that financial support was not received for this work and/or its publication.”

The funder Anhui Province Graduate Education Quality Engineering Project, Grant No. 2023yjsxssfkc027 to Zhi Li, was erroneously omitted.

The correct **Funding** statement appears below:

“The author(s) declare that financial support was received for the research, authorship, and/or publication of this article. This work and/or its publication was supported by the Anhui Province Graduate Education Quality Engineering Project (Grant No. 2023yjsxssfkc027), awarded to Zhi Li.”

The original version of this article has been updated.

